# Carbonate Chemistry and the Potential for Acidification in Georgia Coastal Marshes and the South Atlantic Bight, USA

**DOI:** 10.1007/s12237-023-01261-3

**Published:** 2023-09-11

**Authors:** Janet J. Reimer, Patricia M. Medeiros, Najid Hussain, Stephen F. Gonski, Yuan-Yaun Xu, Ting-Hsuan Huang, Wei-Jun Cai

**Affiliations:** 1 School of Marine Science and Policy, University of Delaware, Newark/Lewes, DE USA; 2 Department of Marine Sciences, University of Georgia, Athens, GA USA; 3 Planetary Technologies, Dartmouth, NS Canada; 4 Department of Oceanography, National Sun Yat-Sen University, Kaohsiung, Taiwan; 5 Mid-Atlantic Regional Council on the Ocean, PA, USA

**Keywords:** Carbonate chemistry, Coastal acidification, *p*CO_2_, Total alkalinity, Dissolved inorganic carbon

## Abstract

In coastal regions and marginal bodies of water, the increase in partial pressure of carbon dioxide (*p*CO_2_) in many instances is greater than that of the open ocean due to terrestrial (river, estuarine, and wetland) influences, decreasing buffering capacity and/or increasing water temperatures. Coastal oceans receive freshwater from rivers and groundwater as well as terrestrial-derived organic matter, both of which have a direct influence on coastal carbonate chemistry. The objective of this research is to determine if coastal marshes in Georgia, USA, may be “hot-spots” for acidification due to enhanced inorganic carbon sources and if there is terrestrial influence on offshore acidification in the South Atlantic Bight (SAB). The results of this study show that dissolved inorganic carbon (DIC) and total alkalinity (TA) are elevated in the marshes compared to predictions from conservative mixing of the freshwater and oceanic end-members, with accompanying pH around 7.2 to 7.6 within the marshes and aragonite saturation states (Ω_Ar_) <1. In the marshes, there is a strong relationship between the terrestrial/estuarine-derived organic and inorganic carbon and acidification. Comparisons of pH, TA, and DIC to terrestrial organic material markers, however, show that there is little influence of terrestrial-derived organic matter on shelf acidification during this period in 2014. In addition, Ω_Ar_ increases rapidly offshore, especially in drier months (July). River stream flow during 2014 was anomalously low compared to climatological means; therefore, offshore influences from terrestrial carbon could also be decreased. The SAB shelf may not be strongly influenced by terrestrial inputs to acidification during drier than normal periods; conversely, shelf waters that are well-buffered against acidification may not play a significant role in mitigating acidification within the Georgia marshes.

**Supplementary Information:**

The online version contains supplementary material available at 10.1007/s12237-023-01261-3.

## Introduction

Increases in global open ocean carbon dioxide partial pressure (*p*CO_2_), the primary driver of ocean acidification (OA; Sabine et al. 2004; Fay and McKinley 2013) due to anthropogenic influences, have been well documented and are keeping pace with the global atmospheric increase of ~2 µatm year^−1^ (McKinley et al. 2011; Bates et al. 2012, 2014; Wanninkhof et al. 2013; Fay and McKinley 2013). In coastal regions and marginal bodies of water, however, the *p*CO_2_ increase in many instances is greater than that of the open ocean due to terrestrial inputs of CO_2_, decreasing buffering capacity and/or increasing water temperatures (Tseng et al. 2007; Thomas et al. 2007; Shadwick et al. 2010; Reimer et al. 2017a; Cai et al. 2021). Coastal oceans receive freshwater from rivers and groundwater as well as terrestrial-derived organic matter, all of which have a direct influence on coastal carbonate chemistry (Jiang et al. 2013; Hu et al. 2015; Salisbury and Jonsson 2018; Brodeur et al. 2019). Understanding the terrestrial influence, including marsh, river, and estuarine-derived material, as well as river stream flow volume, on coastal and near-shore *p*CO_2_ and carbonate chemistry should provide better insights into the mechanistic biogeochemical and physical drivers that affect long-term changes in coastal ocean *p*CO_2_ and subsequently coastal OA (Alin et al. 2012; Gledhill et al. 2015; Xue et al. 2016, 2017).

Elevated total dissolved inorganic carbon (DIC) and low total alkalinity (TA) transported from coastal regions could be an important source of seasonal to decadal scale *p*CO_2_ changes along parts of the US east coast, including the Georgia coast of the South Atlantic Bight (SAB) (Jiang et al. 2008b, 2013; Reimer et al. 2017a). DIC and TA from Georgia coastal marshes during 2005 and 2006 contributed to elevated *p*CO_2_ across the shelf (Jiang et al. 2013) and, therefore, presumably decreased pH. Previous works have also demonstrated large DIC, TA, and *p*CO_2_ variations occur in Georgia coastal marshes (Cai and Wang 1998) and that DIC and TA transport offshore is an important driver of shelf carbonate system variability (Wang and Cai 2004; Xu et al. 2020), although these studies have not elaborated on the current state of acidification within the marshes. Additionally, studies on the inner shelf of the SAB, off the coast of Savanna, Georgia, using just over 1 year of continuous *p*CO_2_ time series from the Gray’s Reef mooring have shown, using one-dimensional biogeochemical models, that transport is an important mechanistic driver for offshore carbonate system variability (Xue et al. 2016, 2017). Decomposition of dissolved organic carbon (DOC), in transit across the SAB, has been shown to contribute to the DIC pool (Jiang et al. 2010, 2013) and is an important mechanism in the overall carbon cycle, since transport from rivers contributes to offshore DOC (Medeiros et al. 2016).

An inner shelf (<20 m isobath) *p*CO_2_ time series in the SAB shows that non-thermal sources of *p*CO_2_ variability, thus carbonate chemistry, are the likely cause of sub-decadal to multi-decadal trends (Sutton et al. 2011; Xue et al. 2016; Reimer et al. 2017a, b). Long-term decreases in pH, which are controlled by carbonate chemistry variables, on the SAB shelf have been estimated to range from −0.003 to −0.004 units year^−1^ (Reimer et al. 2017a). The terrestrial influence on coastal SAB carbonate chemistry and acidification, however, is not well defined. Several studies have specifically shown that there is seasonal terrestrial influence on carbonate variables at the Gray’s Reef mooring (Xue et al. 2016, 2017), though the extent to which terrestrial influence affects acidification across the SAB is still unclear, largely due to a lack of data synthesis. Global climate change is known to directly influence weather patterns in the Southeast USA via shifts in North Atlantic Oscillation and the Bermuda High, which can bring anomalously high rainfall to the region (Intergovernmental Panel on Climate Change 2018). The quantity of marsh-derived dissolved organic matter (DOM) transported from Georgia marshes to the coastal zone and near-shore regions is known to increase due to enhanced precipitation or increased river discharge (Letourneau and Medeiros 2019). Therefore, understanding how terrestrial-derived carbon influences carbonate chemistry in the coastal marshes and SAB shelf could give insights into coastal OA.

In general, SAB waters are well-buffered against acidification due to the characteristic warm salty waters (Egleston et al. 2010; Cai et al. 2020). The coastal waters that surround the SAB, specifically those along the coast of Georgia, are salt marsh-dominated estuaries that are important habitat for foundation species, including oysters and *spartina* grasses (Crotty et al. 2018; Thompson et al. 2020). The SAB shelf is a wide shelf region off the US east coast that is influenced by carbon inputs and freshwater from 10 major rivers, of which the Altamaha is the largest, and coastal marshes that stretch from Cape Canaveral, Florida to Cape Hatteras, North Carolina (Wang and Cai 2004; Cai et al. 2006; Jiang et al. 2010; Wang et al. 2013). The core of Gulf Stream is largely located offshore, with infrequent eddies or intrusions onto the middle and inner shelves (Castelao 2011, 2014). DOM from the Altamaha River can be exported to the SAB shelf break during enhanced river discharge (Medeiros et al. 2017). Therefore, a better understanding of how marsh-driven carbonate chemistry impacts coastal and shelf waters is important for predicting how OA may impact the SAB region in a changing climate. In this region, the terrestrial-derived organic matter pool found in the coastal zone is likely highly dominated by marsh-derived organic materials due to high productivity within the marshes (Hopkinson et al. 2012). The objective of this work is to assess carbonate system variability across the land-estuarine-ocean continuum off the coast of Georgia, USA, and determine if there are potential “hot-spots” for acidification within the coastal zone and near-shore regions. It is hypothesized that while SAB shelf water is well-buffered, coastal waters landward of the 20 m isobath (inner shelf and coastal regions, Jiang et al. 2013; Reimer et al. 2017b; Xu et al. 2017) are more acidic than shelf waters due to DIC produced by the respiration of terrestrial and marsh-derived organic matter and low buffering capacity from freshwater inputs.

## Methods

**Fig. 1:**
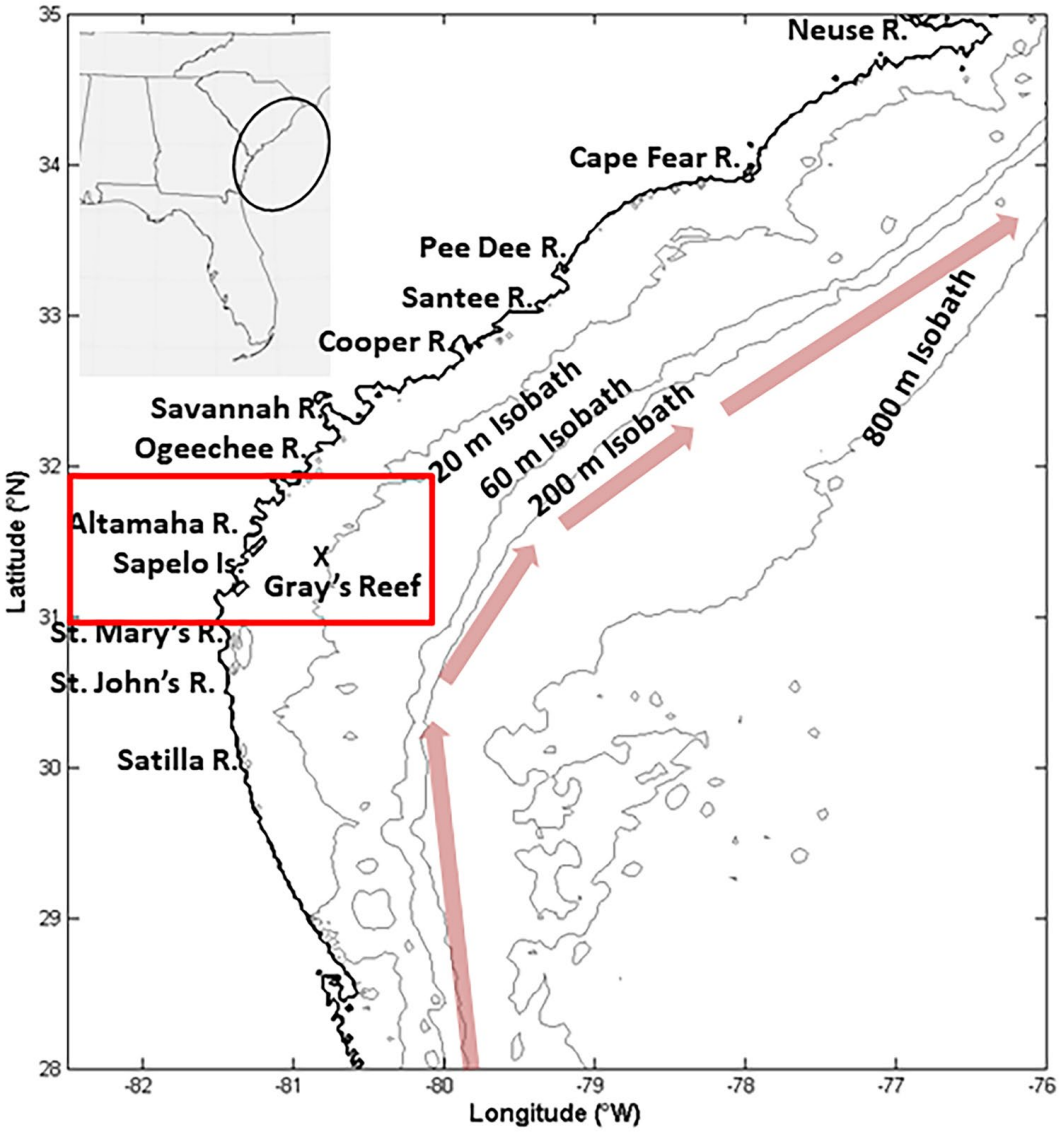
The location of the South Atlantic Bight (SAB) in the Southeast USA, insert, and the 10 major rivers that flow into the SAB. The focus of this study, the central Georgia Bight, is located within the red square. Within the study region, the Gray’s Reef *p*CO_2_ time series is indicated by the black X and is located just landward of the 20 m isobath, traditionally the seaward limit of the inner shelf. The red arrows indicate the mean location of the core of the Gulf Stream. Sapelo Island is also noted here, within the red box of the study area

During 2014, carbonate chemistry variables were measured during  six cruises (April, May, July, September, November, and December) aboard the *R/V Savannah* across the central SAB (Georgia Bight) off the coast of Georgia, USA. All data used in this study were collected as part of the Georgia Coastal Ecosystems Long-term Ecological Research (GCE-LTER) project. Observations were collected over 24-h time periods while underway. Note that in an earlier study, diurnal *p*CO_2_ variations were approximately 20 µatm in the offshore waters (Jiang et al. 2008a, b); however, diurnal differences in near-shore systems are expected to be larger. The cruises concentrated on the inner shelf of the SAB and the marshes, though did extend as far as the shelf break and the 200 m isobath (seaward extent of the outer shelf) once during each cruise. All cruises passed the mouth of the Altamaha River, which is the largest watershed in the region and empties into the coastal marshes near Sapelo Island, GA at ~31.5°N (Fig. 1). As a freshwater source, the Altamaha is the third largest freshwater source to the eastern shore of North America (Schaefer and Alber 2007). The extensive wetlands, including tidal saltmarshes, of the lower Altamaha River contribute over half of the total organic carbon delivered to the coastal zone of the Georgia Bight, primarily due to the extensive, and increasing, agricultural activity (Weston et al. 2009). River discharge in the lower Altamaha River is routinely measured by the U.S. Geological Survey (USGS) at Doctortown, GA (available at http://waterdata.usgs.gov) above the head of the tide. River discharge data from 2000 through 2018 were used to calculate the climatology. Daily mean river stream flow was calculated from 15-min frequency observations.

Discrete surface water samples were collected during the cruises for DIC, TA, pH_NBS_ (pH measured on the National Bureau Standards scale), salinity, temperature, DOC, and DOM composition from the coastal zone, including the mouth of the Altamaha River, across the shelf out to the mean location of the core of the Gulf Stream, and also sampling around the Gray’s Reef *p*CO_2_ time series (Fig. 1). Salinity and temperature of the discrete TA and DIC samples were measured by the R/V Savannah’s CTD (SBE 911) mounted on the rosette, and the salinity and temperature for underway measurements were measured by a Sea Bird 45. All surface samples were collected at a depth of 0 to 3 m and sampling within the marshes was only conducted at high tide, or shortly before or after, due to draft of the ship. As needed, DIC and TA samples, especially in the intracoastal waters, were filtered directly from the Niskin bottles with an inline 0.45-µm Whatman GF/F cartridge filter. Care was taken to ensure that air was released from the filter and that the filter was properly flooded with the appropriate volume of water prior to sample collection. DIC and TA were treated with saturated mercuric chloride, refrigerated, and transported to the laboratory for analysis. Due to the linearity of the TA-salinity relationship in this, and previous studies of Georgia coastal waters (Xue et al. 2016; Reimer et al. 2017a), it is not likely that carbonate system results herein are influenced by suspended carbonate sediments. Additionally, the use of the GF/F filter would eliminate any carbonate particles. DIC and TA samples were stored in a refrigerator while onboard and were measured within 2 weeks upon return to the laboratory following the methods of Jiang et al. (2008a) and Huang et al. (2012), with a precision of ±0.1%. All DIC and TA presented herein are from the surface layer. Therefore, variability only reflects biological, surface transport, and air-sea exchange processes; formation/dissolution of calcium carbonate is not represented. DIC, TA, salinity, and temperature were used to calculate the aragonite mineral saturation state (Ω_AR_) and Revelle factor, sensitivity to changes in DIC and TA, using the Matlab version of CO2SYS (Lewis and Wallace 1998), with disassociation constants by Merhbach refit by (Dickson and Millero 1987).

Samples for pH_NBS_ were collected from each Niskin bottle in 125-mL Pyrex bottles and brought to 25 °C in a water bath and measured within 2 h of collection on board the ship. The samples remained in the water bath while waiting to be analyzed. Samples were read with an Orion Ross combination glass electrode that was calibrated with NBS 4.01, 7.00, and 10.01 buffers for a multipoint calibration.

DOC and DOM were collected at a sub-set of cruise stations during each cruise, except December. Water samples were collected using Niskin bottles mounted on a CTD rosette into pre-cleaned Nalgene carboys. In the ship laboratory, samples were filtered through pre-rinsed 0.2-µM Pall Supor membrane filters, and filtrates were then collected into (1) 60-mL amber Nalgene bottles and immediately frozen (at –20 °C) until DOC concentration analysis and (2) acid-washed 1-L polycarbonate bottles, acidified to pH 2 using concentrated HCl and refrigerated at 4 °C. Once in the laboratory onshore, DOM was extracted from the acidified filtrates using solid-phase extraction (SPE) cartridges (Agilent Bond Elut PPL) following Dittmar et al. (2008), and subsequently eluted with methanol. The methanol extracts were then kept frozen (−20 °C) and in the dark until molecular composition analysis. DOC concentrations were measured in water samples using a Shimadzu TOC-L_CPH_ with daily potassium hydrogen phthalate (KHP) standard curves and regular analysis of Consensus Reference Materials (Hansell 2005). Ultrahigh-resolution mass spectrometry (FT-ICR MS) was used to analyze the molecular composition of the DOM extracts using a Fourier transform-ion cyclotron resonance mass spectrometer with electrospray ionization (negative mode). Detailed information about the data processing is given by Medeiros et al. (2017). DOM composition was used to determine an index indicative of riverine inputs, *I*_Terr_, based on the ratio of the relative abundances of molecular formulae that were strongly correlated with δ^13^C signatures. The ratio is *I*_Terr_ = *Terr*/(*Terr* + *Mar*), where *Mar* and *Terr* are the sum of the FT-ICR MS signal intensity of 40 molecular formulae that are strongly correlated positively and negatively with δ^13^C signatures, respectively. The ratio increases with the riverine character of the DOM and has been found to be strongly correlated positively with other indicators of terrigenous input (Medeiros et al. 2016).

Continuous surface underway data, including *p*CO_2_ (Apollo SciTech [AS-P2] with a LI-COR LI-7000), salinity, and temperature (SBE-45 thermosalinograph), were also collected during the six cruises in 2014. *p*CO_2_ calibrations occurred approximately every 6 h using a multipoint calibration with NOAA-certified standards with concentrations of 151.5, 395.4, and 1969 ppm CO_2_. The method was documented and compared with a NOAA system in earlier work (Jiang et al. 2008b). Underway *p*CO_2_, sea surface temperature (SST), and sea surface salinity (SSS) observations are at 2-min intervals. *p*CO_2_ and supporting information can be obtained from the Surface Ocean Carbon Atlas version 5 release (SOCAT; https://doi.pangaea.de/10.1594/PANGAEA.877863). In this study, however, we exclude September *p*CO_2_ due to poor data quality.

## Results and Discussion

### River Stream Flow and TA and DIC Mixing Behaviors

**Fig. 2:**
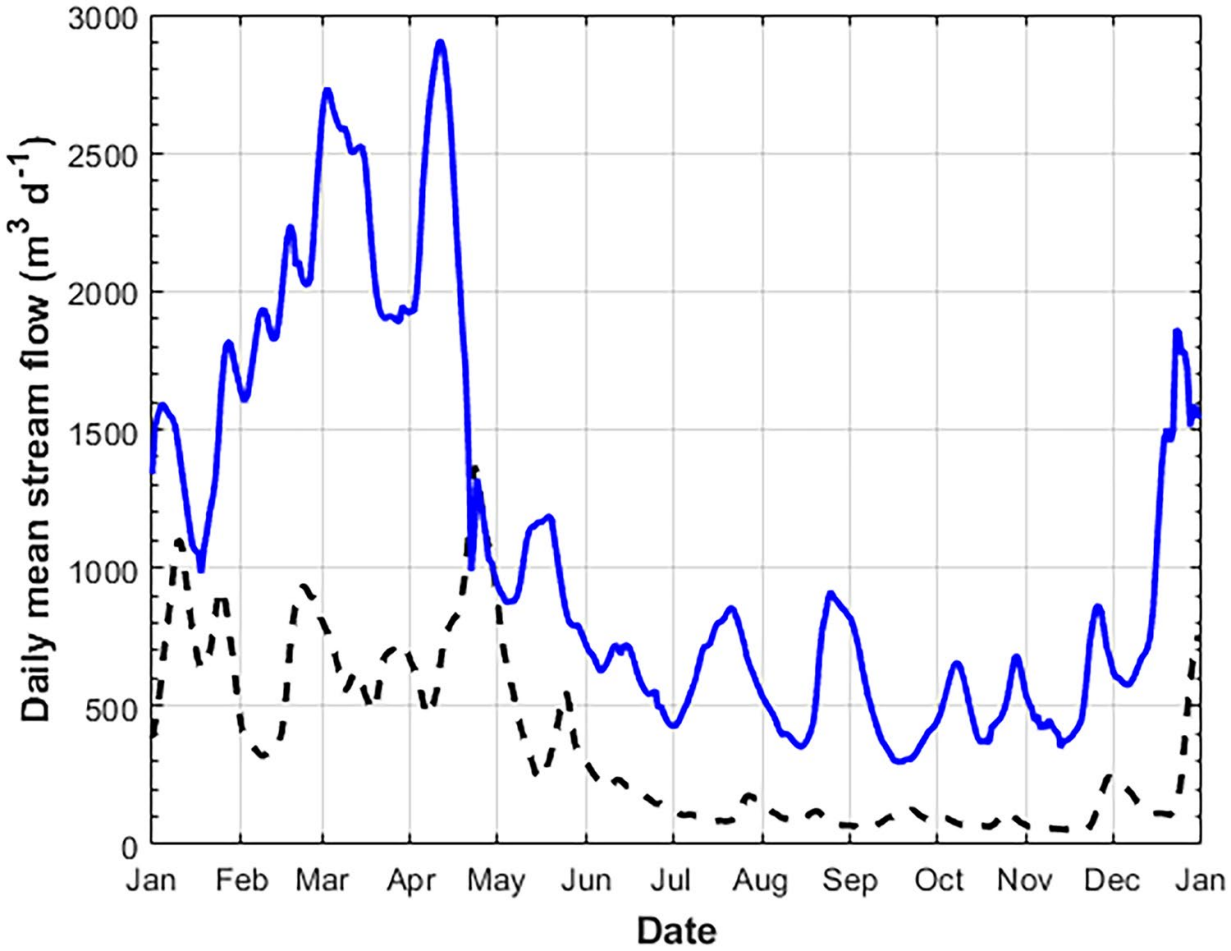
Altamaha River stream flow from 2014 (black dashed line) and climatological mean river stream flow from 2001 through 2014

The Georgia coastal zone is greatly influenced by freshwater sources (Menzel 1993). Altamaha River stream flow is typically increased during the winter into the middle of the spring, generally from December to May (Fig. 2). Therefore, it is expected that TA and DIC will have a strong dependence on salinity and the amount of freshwater entering the coastal zone. During 2014, however, river stream flow was anomalously low compared with the climatological mean, likely due to shifts in the Bermuda High climate index and/or North Atlantic Oscillation that can periodically cause dry conditions in the Southeast USA. Low precipitation throughout the watershed and decreased river stream flow occurred during the 2014 calendar year (Sheldon and Burd 2014), while 2014 river stream flow followed the typical climate pattern, increased in the winter, and decreased in the summer; winter highs and summer lows were both below average. Therefore, it can be assumed that the flux of dissolved and particulate matter from the river and marsh would also be reduced during this dry climate period. Even though 2014 was drier than normal, and contrary to long-term predicted climate patterns in the Southeast USA (IPCC 2018), an assessment of carbonate chemistry variability under dry conditions could provide a conservative estimation of how coastal freshwater and marshes likely impact shelf carbonate chemistry conditions.

**Fig. 3:**
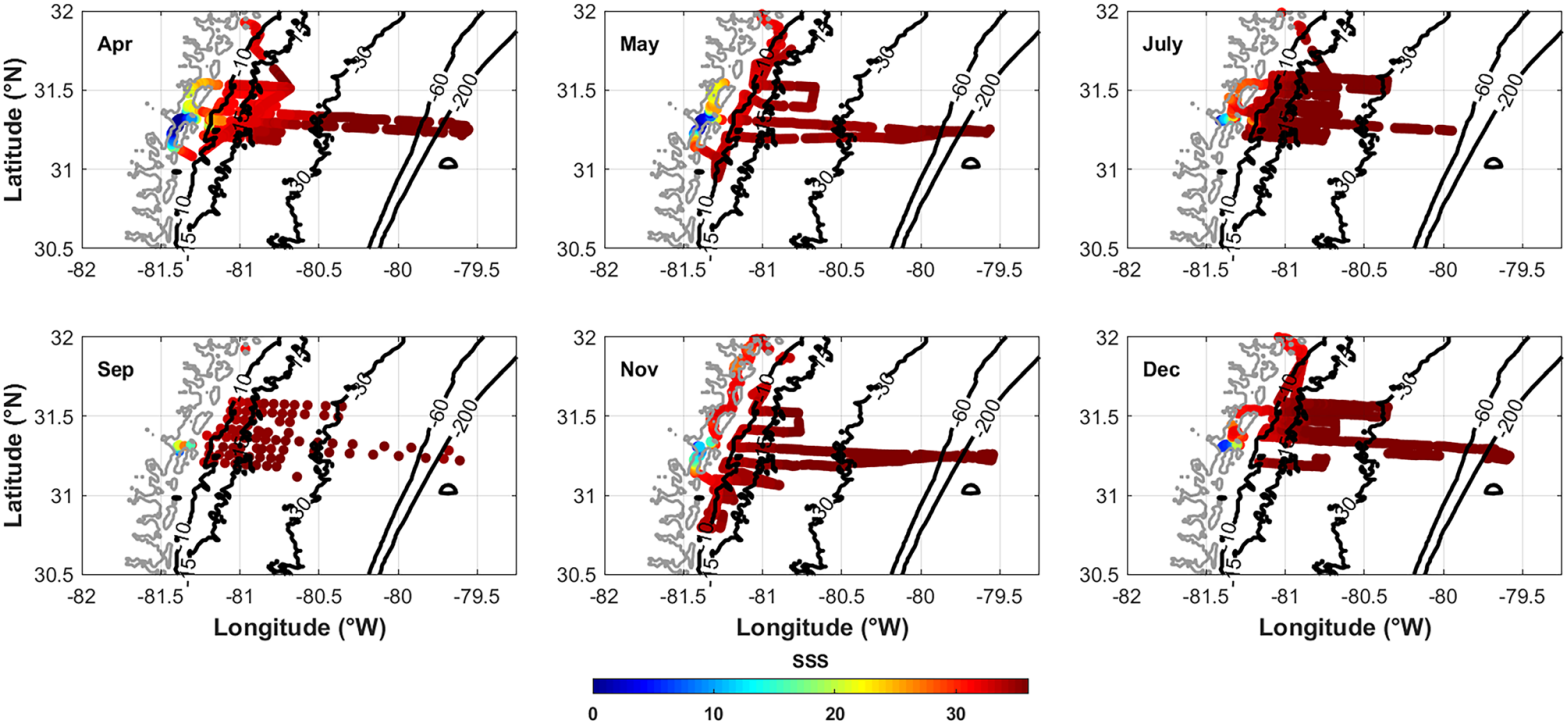
Sea surface salinity spatial distribution for each of the six cruises in 2014. Salinity generally increases across the shelf away from freshwater sources. The furthest distribution of the lower salinity plume from the marshes onto the shelf occurred in April

River stream flow has a direct impact on coastal zone surface salinity as lower salinity waters extend farther offshore during wetter periods (Fig. 3) (Xue et al. 2016), as in April and May when river stream flow is increased (Fig. 2). The mixing of low-salinity riverine water and shelf oceanic waters was shown to be an important driver of dissolved inorganic and organic carbon and particulate matter transport on to the SAB shelf (Jiang et al. 2013; Signorini et al. 2013; Xue et al. 2016, 2017), specifically from April to May 2014 where wind-driven mixing was found to be the dominant driver (Medeiros et al. 2017). By July, however, the low-salinity plume that occurred in April and May was again held within the inner shelf (Fig. 3) when the river stream flow decreased.

**Fig. 4:**
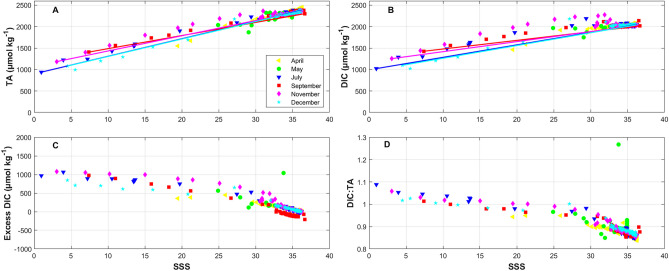
**A** TA conservative mixing lines for each cruise. **B** DIC conservative mixing lines for each cruise. **C** Excess DIC (eDIC) versus salinity for each cruise. Here, eDIC is defined as the excess of observational data relative to the mixing line. eDIC decreases offshore away from the inputs from the marshes. **D** DIC:TA ratios versus salinity for each cruise. The DIC:TA decreases offshore

The linearity of the TA and DIC to SSS suggests a strong dependence on mixing behavior across the salinity continuum; however, DIC is less linear than TA (Table 1). SSS explains greater than 94% of the variability in TA, except November, and about 90% of the variability in DIC, except May and November (Table 1). In November, only 63% of TA and 58% of DIC variability was explained by salinity with both parameters in the mid-SSS range 200 to 400 µmol kg^−1^ above the conservative mixing line (Fig. 4A, B). Elevated TA and DIC, or excess DIC (eDIC), likely indicate sources in this portion of the marsh (Fig. 4C), which will be discussed further in the next section, coming from within the tidal creeks of the marsh that drain at high tide (Cai and Wang 1998; Cai et al. 2021; Wang et al. 2018). DIC to TA ratios (DIC:TA) decrease offshore (Fig. 4D) and will be discussed below. The spatial sampling inconsistencies, such as no sampling in the freshwater regions in May, from cruise to cruise, could bias any inter-seasonal comparisons of TA-SSS and DIC-SSS mixing; therefore, caution should be used when trying to determine any time-based comparisons. The strong dependence of TA and DIC on SSS directly impacts the distribution of the other carbonate system parameters. There could be additional bias when comparing diel and/or tidal differences as well, since samples were collected during both day-time and night-time hours, on the shelf and in the coastal zone. In general, as seen in Fig. 4, both TA and DIC are highly conservative, with lower concentrations in fresher coastal waters than offshore. Earlier studies have shown generally higher DIC and TA values during lower tide periods, indicating HCO_3_^−^ sources from tidal marshes, but not specifically related to diel light cycles (Cai and Wang 1998; Wang and Cai 2004). Therefore, when considering the diel biases, there is little evidence within this dataset that time of sampling greatly impacts spatial highs and lows, though in future studies this should be considered.

**Table 1:** TA-SSS and DIC-SSS relationships during the six cruises. The numbers in the parentheses are the standard error of the constants

**Month**	**Equation**	***p*-value;** ***r***^**2**^**-value;** **number of samples**
**April**	TA = 48.7(±0.9) × SSS + 673(±30)	<0.001; 0.99; 43
**May**	TA = 42.2(±1.5) × SSS + 838(±48)	<0.001; 0.94; 51
**July**	TA = 37.4(±0.4) × SSS + 1032(±12)	<0.001; 0.99; 102
**September**	TA = 31.3(±0.4) × SSS + 1217(±13)	<0.001; 0.97; 93
**November**	TA = 28.3(±2.9) × SSS + 1373(±93)	<0.001; 0.63; 61
**December**	TA = 42.5(±0.5) × SSS + 831(±8.0)	<0.001; 0.99; 70
**April**	DIC = 30.1(±1.7) × SSS + 1009(±55)	<0.001; 0.89; 43
**May**	DIC = 18.7(±10) × SSS + 411(±342)	ns; 0.08; 51
**July**	DIC = 22.7(±0.7) × SSS + 1231(±25)	<0.001; 0.91; 102
**September**	DIC = 17.7(±0.7) × SSS + 1403(±22)	<0.001; 0.88; 93
**November**	DIC = 16.5(±2.0) × SSS + 1489(±66)	<0.001; 0.58; 61
**December**	DIC = 30.3(±0.9) × SSS + 975(±32)	<0.001; 0.93; 70

### Spatio-Temporal Distribution of TA, DIC, pH, and *p*CO_2_

**Fig. 5:**
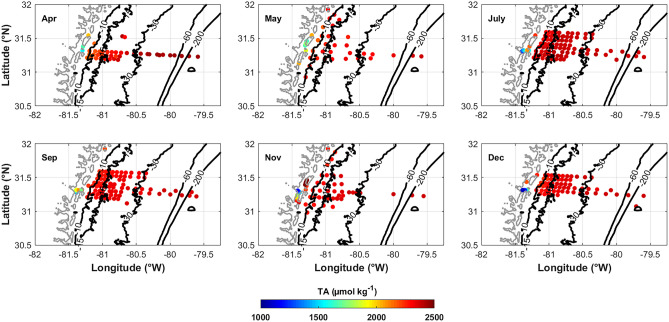
Surface TA spatial distribution for each of the six cruises in 2014. TA generally increases across the shelf away from the freshwater sources of the coastal zone; however, there are “hot-spots” within the marshes

**Fig. 6:**
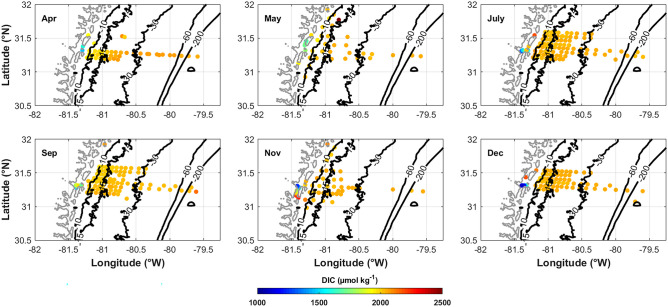
Surface DIC spatial distribution for each of the six cruises in 2014. DIC generally increases across the shelf away from the freshwater sources of the coastal zone; however, there are “hot-spots” within the marshes

**Fig. 7:**
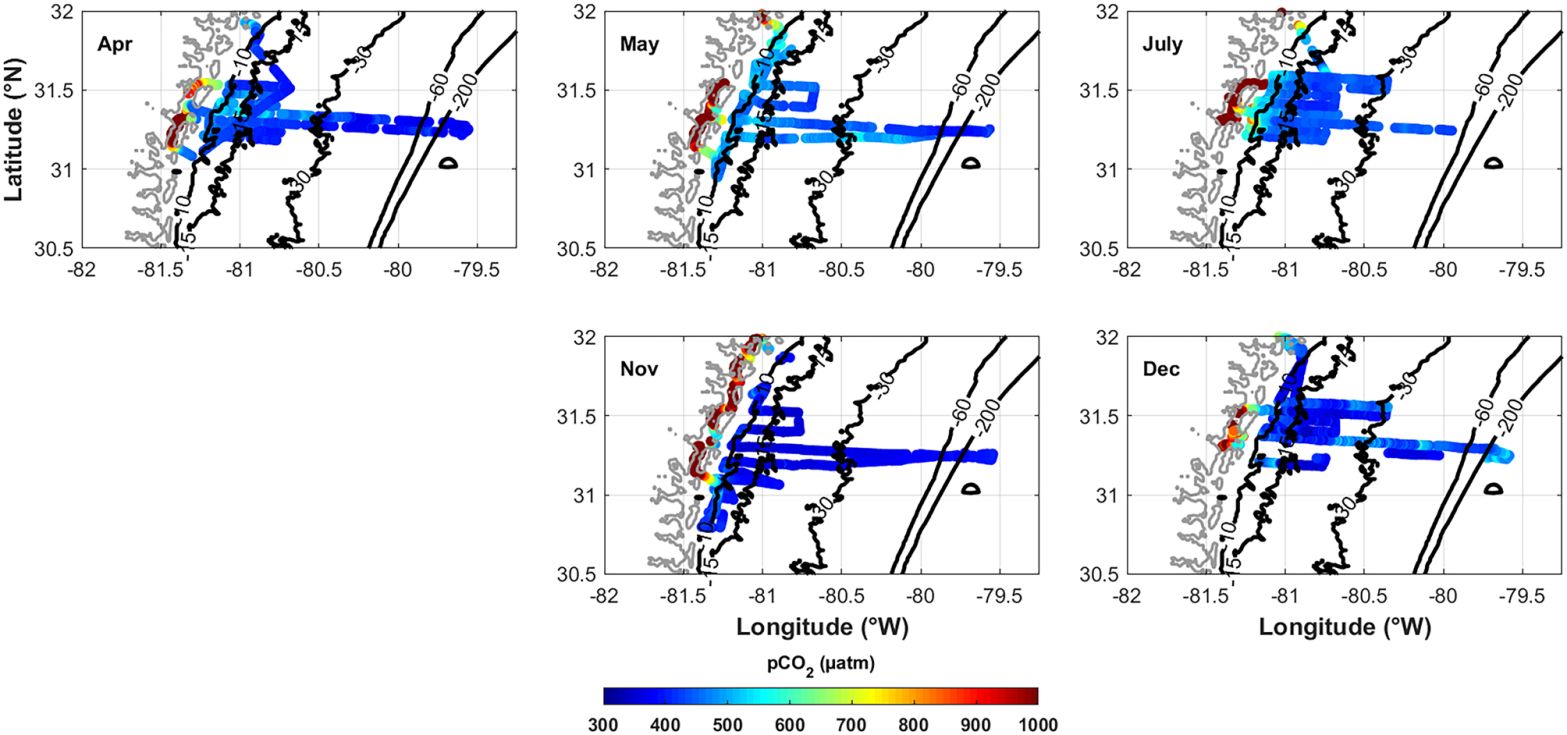
*p*CO_2_ spatial distribution for each of the six cruises in 2014. *p*CO_2_ generally decreases across the shelf away from the freshwater sources of the coastal zone

**Fig. 8:**
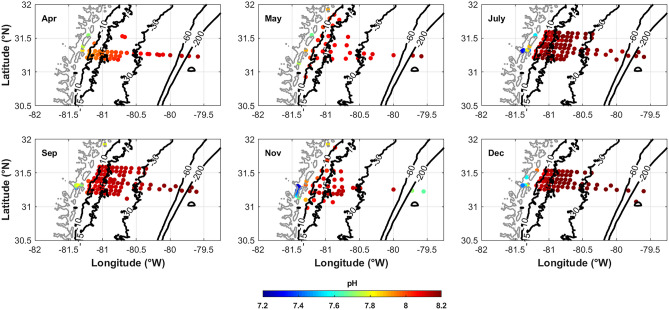
pH spatial distribution for each of the six cruises in 2014. pH generally increases across the shelf away from the freshwater sources of the coastal zone

TA (Fig. 5) and DIC (Fig. 6) in the lowest salinity portions of the marshes are generally decreased with respect to concentrations on the shelf. TA ranges from approximate 990 µmol kg^−1^ at a salinity of 1, in July in the mouth of the Altamaha River, to > 2400 µmol kg^−1^ at a salinity of 36.6 on the outer SAB shelf. DIC follows a slightly different pattern, still with the lowest values in the lowest salinity portion but the highest values in the brackish salinity range (~25 to 32) within the marsh and near-shore (shallower than 15 m), and then slightly decreases in higher salinity waters on the shelf. Relative to river end-member values, TA and DIC are all elevated inside the marshes. In particular, DIC is more elevated than TA (Fig. 4A vs B). Generally, since the DIC to TA ratio (DIC:TA) controls *p*CO_2_ and pH, when DIC is elevated compared to TA (high DIC:TA values), *p*CO_2_ increases, which explains why underway *p*CO_2_ is consistently elevated in the marshes compared to the shelf (Fig. 7). The same scenario also causes pH decreases, which are seen in the marshes relative to the shelf (Fig. 8). TA and DIC temporal evolution follows the low-salinity plume pattern from April through July, where decreased values extended farther offshore in April than May (Figs. 5 and 6). By July, the plume had dissipated. DIC:TA ratios are consistently <1 throughout the year though ~1 within the marshes, which is likely due to sulfate reduction in the marshes where there is high organic matter content (Cai and Wang 1998).

In general, *p*CO_2_ decreases across the shelf as the elevated values from the marsh mix with ocean water (Jiang et al. 2008b, 2013) and buffering capacity, driven by increased TA, increases (Egleston et al. 2010). Diurnal differences can also be seen across the shelf on the longer transects, with increased (cyan-blue) values occurring at night (*p*CO_2_ due to respiration) and decreased (violet-blue) values occurring during the day (*p*CO_2_ uptake for photosynthesis). Previous studies in the SAB have also concluded that *p*CO_2_ from the marshes is transported across the shelf (Jiang et al. 2008b, 2013; Xue et al. 2016). A clear temporal *p*CO_2_ pattern is difficult to discern due to the spatial inconsistency of sampling within the marsh (Fig. 7). Between April and May, there is a *p*CO_2_ increase on the shelf consistent with the low-salinity plume, which was attributed to wind-driven offshore mixing (Medeiros et al. 2017). *p*CO_2_ on the shelf in July remained relatively elevated, compared to November and December, consistent with respiration of the terrigenous DOM that was found out to the shelf break (approximately −79.9°W; Medeiros et al. 2017). In November and December, *p*CO_2_ decreases with temperature. It should be noted that in December, warmer Gulf Stream waters encroached as far as ~30 m isobath and cause a slight increase in *p*CO_2_ (Fig. S1).

Since the DIC:TA value controls the pH, where DIC:TA values are higher, the pH is decreased, as is the case in the marshes. Specifically, in the lowest salinity regions, pH can be as low as 7.2 with a DIC:TA value as high as almost 1.1 (Figs. 4D and 8). pH then increases across the shelf, up to 8.2, as DIC:TA decreases to around 0.85 (Figs. 4D and 8). Temporally, pH is lowest in April, at the end of the wet season when freshwater influx to the marshes was enhanced. In April, the decreased pH extends farther offshore than any of the other months sampled (Fig. 8). Low-pH freshwater plumes, where DIC:TA is increased, are often noted along the east coast of the USA and can have harmful effects on biota (Salisbury et al. 2009; Hu et al. 2015; Salisbury and Jonsson 2018).

### Mechanistic Drivers of *p*CO_2_ Variability

**Fig. 9:**
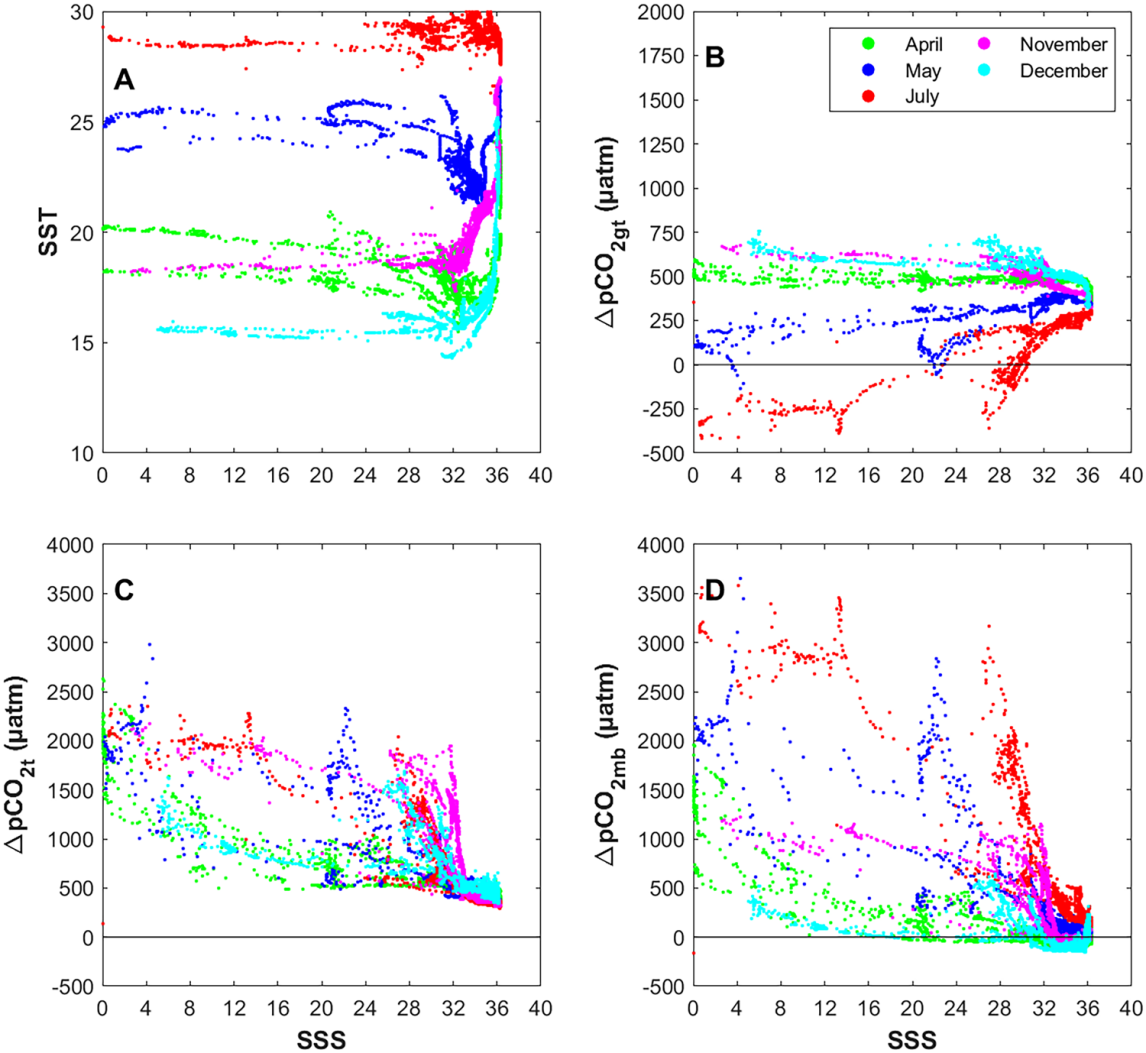
Salinity versus SST (**A**) and the mechanistic drivers of changes in *p*CO_2_: ∆*p*CO_2gt_ (**B**), ∆*p*CO_2t_ (**C**), and ∆*p*CO_2nt_ (**D**)

To understand the drivers of *p*CO_2_ variability, we applied a one-dimensional (1D) mechanistic calculation to decompose the changes due to thermal influences, air-sea exchange, and biological and mixing processes (Jiang et al. 2008a; Xue et al. 2016). First, *p*CO_2t_ is calculated as:

1 $$p{\mathrm{CO}}_{2\left(\mathrm{meanSST}\right)}={p{\mathrm{CO}}_2}^{0.0412\times\left(\mathrm{meanSST}-\mathrm{SST}\right)}$$

where *p*CO_2(meanSST)_ is *p*CO_2_ adjusted to the mean SST of 22.29 °C (determined from all SST observed from all cruises in 2014; Fig. 9A), with the value of the exponent determined specifically for the SAB (Reimer et al. 2017b). Therefore, the change in surface *p*CO_2_ due to thermal influences (∆*p*CO_2t_; Fig. 9B) is:

2 $$\Delta p{\mathrm{CO}}_{2\mathrm{t}}=p{\mathrm{CO}}_{2}-p{CO}_{2\mathrm{meanSST}}$$

Next, the change in *p*CO_2_ due to gas exchange, which includes a thermal component (Δ*p*CO_2gt_; Fig. 9C), is calculated via the equation:

3 $$\Delta p{\mathrm{CO}}_{2\mathrm{gt}}=p{\mathrm{CO}}_{2}-(\Delta p{\mathrm{CO}}_{2\mathrm{t}}+\Delta p{\mathrm{CO}}_{2\mathrm{g}})$$

where Δ*p*CO_2g_ is the change in *p*CO_2_ due to the air-sea difference (sea minus air), which uses the 2014 mean atmospheric *p*CO_2_, 396.8 ± 10.8 µatm, from the Gray’s Reef acidification mooring located within the study region (Reimer et al. 2017a). Here, the arithmetic mean is used due to large gaps in the local dataset for 2014, rather than using the daily observed value for each iteration. Negative values represent oceanic uptake. By further calculation, the combined variation due to non-thermal (biological and mixing) processes (∆*p*CO_2nt_; Fig. 9D) term is:

4 $$\Delta p{\mathrm{CO}}_{2\mathrm{nt}}=p{\mathrm{CO}}_{2}-p{\mathrm{CO}}_{2\mathrm{gt}}$$

The SST normalization uses the mean SST from all the cruise data collected (22.29 ± 4.51 °C); however, this could lend itself to large uncertainties in Eqs. 1 through 4 up to 18.04%. Additionally, the ~2.7% uncertainty in the mean atmospheric *p*CO_2_ adds to the absolute value of uncertainty in the mechanistic calculations. This estimate should be considered a first approximation of the general behavior of various mechanistic drivers and needs to be refined in future efforts.

In general, ∆*p*CO_2t_ decreases offshore (increased salinity) and is greatest during the warmer periods (May day-time transect, July, and November). Offshore, ∆*p*CO_2t_ is greatest in November and December, consistent with the increased temperatures associated with the Gulf Stream location (Signorini and McClain 2007; Castelao 2011). The large seasonal change in ∆*p*CO_2t_ and ∆*p*CO_2gt_ from April to May can be explained by the ~5 to 7 °C temperature increase (Fig. 9A**)**. ∆*p*CO_2gt_ was negative in the marsh and coastal zone during July likely due to *p*CO_2_ uptake by plants and phytoplankton in the marshes during the most productive season when DOC and chlorophyll *a* are highest (Wang et al. 2018). This seasonal change is also evident in the ∆*p*CO_2nt_ values from April to May. During this time, not only did the increase in seasonal warming contribute to increased biological respiration of OM (release of CO_2_ to the water) but increased wind-driven mixing across the shelf from April to May accounts for the large change in ∆*p*CO_2nt_ (Medeiros et al. 2017). During the anomalously dry 2014 period, ∆*p*CO_2nt_ was most important in the marsh and coastal zone, particularly in May, July, and November; however, once the water mass reached the inner shelf, the importance of this driver decreased greatly.

### Terrestrial Contributions to SAB Shelf Carbonate Chemistry Using eDIC and *I*_Terr_ Values

**Fig. 10:**
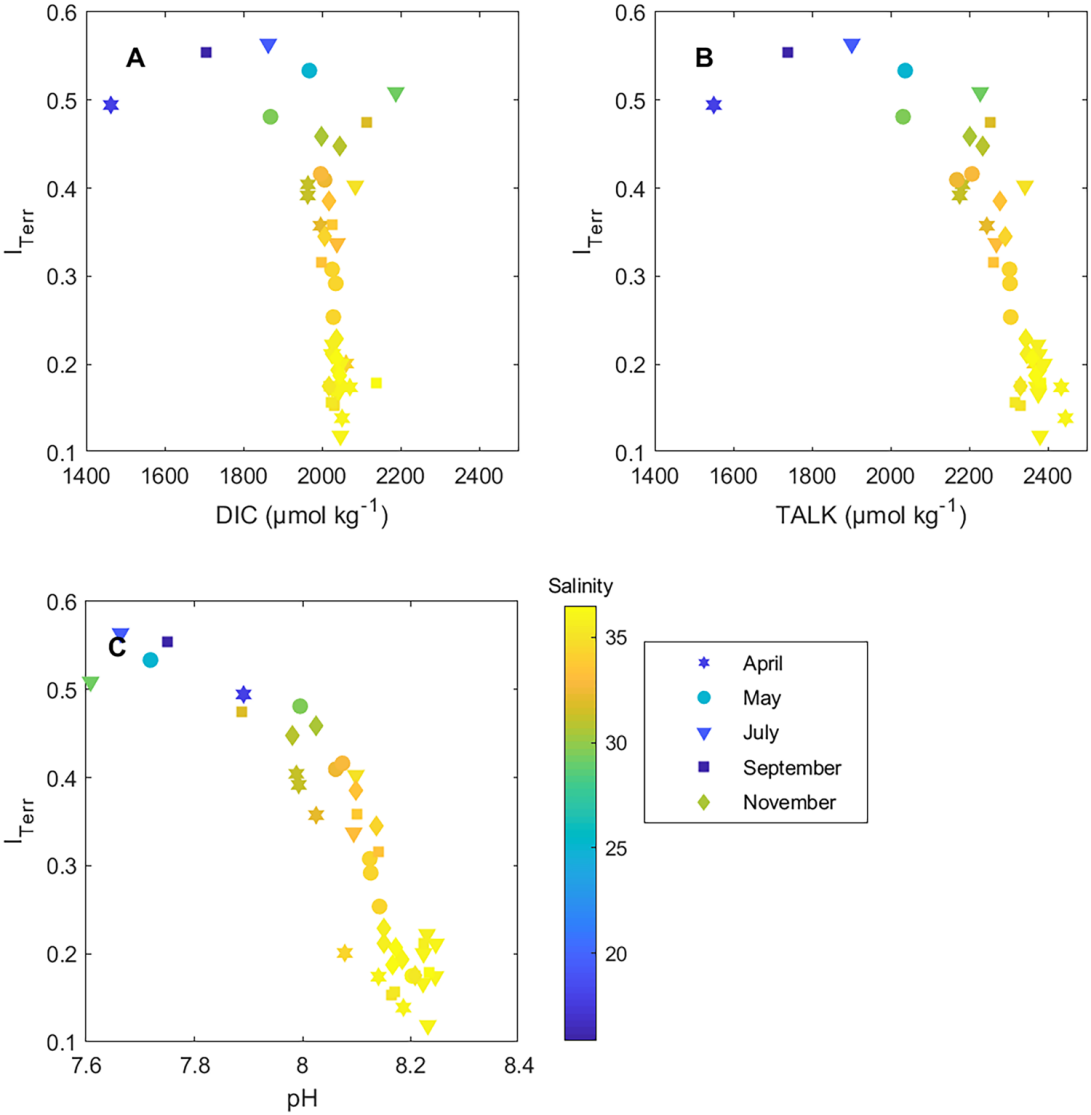
*I*_Terr_ versus **A** DIC, **B** TA, and **C** pH in surface samples. *I*_Terr_ values decrease with increasing salinity, DIC, TA, or pH away from the coastal zone. Greater *I*_Terr_ values indicate greater contributions from terrestrial-derived organic compounds

Previous studies in the SAB have surmised that the offshore transport of OM and *p*CO_2_ from the marshes and coastal zone is important for SAB shelf biogeochemistry or acidification (Signorini et al. 2013; Xue et al. 2016; Reimer et al. 2017a; Sutton et al. 2019). First, to investigate the contribution of terrestrial-derived DIC to the shelf, the calculation of excess DIC (eDIC) can give insights into the quantity of the source of DIC and *I*_Terr_ provides information on the terrestrial/riverine organic matter content along the salinity continuum. Inherently, both eDIC and *I*_Terr_ include contributions from rivers, estuaries, marshes, groundwater discharge, draining from within the tidal channels, and all other non-shelf sources (Jiang et al. 2013; Medeiros et al. 2016). eDIC was calculated as:

5 $$\mathrm{eDIC}={\mathrm{DIC}}_{\mathrm{i}}-\frac{{\mathrm{SSS}}_{\mathrm{i}}}{{\mathrm{SSS}}_{\mathrm{ocean}}}\times {\mathrm{DIC}}_{\mathrm{ocean}}$$

where *i* is the value of DIC or SSS at a given station and ocean indicates the ocean end-member value for either SSS or DIC. For the purposes of this calculation, the ocean end-member is considered the highest salinity value with respective DIC for each individual cruise. Positive values indicate DIC production, or a source of DIC, and negative values indicate that the eDIC value is less than the reference value and could be a sink for DIC (Fig. 4C). It should be noted that this calculation does not identify a mechanistic driver for sources or sinks. The greatest eDIC values (source of DIC) is within the Altamaha River mouth and surrounding marshes (Fig. 4C), which is also where *I*_Terr_ is greatest, suggesting that the terrestrial-derived carbon is being remineralized from organic form to DIC in the marshes and that the DIC at higher salinity is not terrestrial-derived, rather it is of marine origin (Fig. 10A). Unfortunately, DIC and TA samples were not collected in the Altamaha mouth in April or May; therefore, there are no results for zero salinity water at the peak river stream flow in 2014. Overall, eDIC is negatively correlated to salinity and there are even negative eDIC values, indicating a potential DIC loss from the system in shelf waters (Fig. 4C). A similar assessment of eDIC on the SAB shelf in 2005 also found negative correlations to salinity across all seasons (Jiang et al. 2013).

*I*_Terr_, the ratio of the composition of terrestrial organic compounds to terrestrial and marine-derived organic compounds (Medeiros et al. 2017), is weakly to moderately negatively correlated to DIC and TA (Table 2). Therefore, higher DIC and TA values found on the shelf are primarily marine-derived. Previously, it was determined that between April and May 2014, wind-driven mixing across the shelf caused a widening of the dissolved organic matter pool onto the inner shelf from the marshes (Medeiros et al. 2017). Even with the sparse data points in May, DIC is greater within the 10 to 15 m isobath region in May than in April (Fig. 6) and lower salinity DIC is associated with higher *I*_Terr_ values (Fig. 10A), suggesting that the terrestrial-derived organic material that was delivered to the coastal zone from the April river stream flow peak was likely respired to DIC as it was transported across the shelf. Given that 2014 was an anomalously dry year with decreased river stream flow, these conditions may not be a mean representation of future or current SAB carbonate chemistry. Although, these results do show the importance of the relationship between terrestrial-derived organic matter (*I*_Terr_) and DIC and TA (Fig. 10A, B).

**Table 2:** Correlations of *I*_Terr_ (dependent variable) to carbonate system parameters (independent variable)

**Parameter**	**Equation**	***p*-value;** ***r*-value; number of samples**
**DIC**	DIC = −380(±118) × *I*_Terr_ + 2122(±39)	<0.01; 0.45; 43
**TA**	TA = −1038(±133) × *I*_Terr_ + 2576(±44)	<0.001; 0.78; 42
**pH**	pH = −1.038(±0.091) × *I*_Terr_ + 8.401(±0.031)	<0.001; 0.87; 43

*I*_Terr_ is also negatively correlated to pH, with the lowest pH values occurring at the low-end of the salinity range (Fig. 10C), coinciding with the higher *I*_Terr_ values that are associated with terrestrial-derived DOM. In general, rivers have lower pH than ocean water (Salisbury et al. 2009; Cai et al. 2020) and deliver large amounts of OM to the coastal zone, which, once remineralized, can also drive down pH. In the subtropical SAB, however, well-buffered, warm, salty waters (Egleston et al. 2010) likely contribute to the increasing pH away from the coastal zone. Therefore, in the SAB, it is reasonable to hypothesize that marsh and coastal waters, with higher quantities of *p*CO_2_ and lower TA than shelf waters, may be more susceptible to acidification than the inner, middle, and outer shelf regions.

### Acidification in the Marshes and on the SAB Shelf

**Fig. 11:**
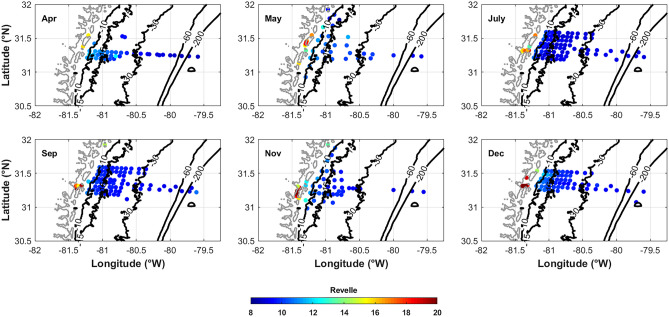
Revelle factor spatial distribution for each of the six cruises in 2014. The Revelle factor generally decreases across the shelf away from the freshwater sources of the coastal zone

**Fig. 12:**
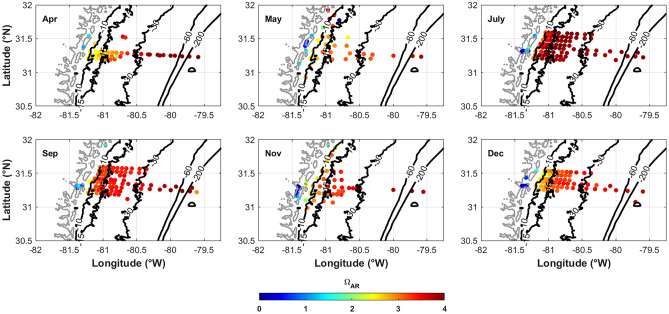
Aragonite saturation state (Ω_Ar_) spatial distribution for each of the six cruises in 2014. The Ω_Ar_ generally increases across the shelf away from the freshwater sources of the coastal zone

Elevated *p*CO_2_, DIC, and *I*_Terr_ coincide with decreased pH and TA along the same gradients, suggesting that the well-buffered shelf waters of the SAB may not have a strong influence on coastal zone carbonate chemistry along the Georgia marshes. Additionally, during low river stream flow periods, such as 2014, the low-pH waters from the coastal zone likely have little impact on the shelf. To determine the potential impact of low-pH waters on biota within the marsh, the aragonite saturation state (Ω_AR_) and Revelle factor (seawater sensitivity to increasing *p*CO_2_) can be used. In 2014, Revelle factor values on the shelf ranged from approximately 10 to 12, similar to other subtropical waters (Egleston et al. 2010), whereas values within the marshes were as high as 20 (Fig. 11). Higher Revelle factor values within the marsh indicate a greater sensitivity to change due to increased *p*CO_2_. Ω_AR_ within the marshes was also <1 (Fig. 12) at the low-salinity sites where TA and pH were lowest, indicating a lower buffering capacity in the fresher waters (Salisbury et al. 2009). In general, the buffering capacity of the SAB is greater than cooler coastal waters at higher latitudes or in coastal upwelling regions (Egleston et al. 2010). The elevated Revelle factor values with Ω_Ar_ values <1 indicate that Georgia marshes could be at greater risk of acidification than the well-buffered shelf waters. Over two decades of CO_2_ and calculated pH on the SAB shelf has shown a slow pH decline, relative to other coastal regions, though faster than the open Subtropical Atlantic (Reimer et al. 2017b). Assessing the seasonal changes in carbonate chemistry and variability related to climate patterns can help predict how acidification will respond to changing climate conditions.

There are “hot-spots” where DIC and TA are elevated within the marsh, though DIC:TA ratios and Ω_Ar_ are still <1 (dark blue in Fig. 11). Wang et al. (2018) determined that the Duplin River, a tidal channel that drains the tidally flooded salt marshes of Sapelo Island into Doboy Sound, exports large quantities of DIC. TA and DIC hot-spots also fall above the conservative mixing lines (Fig. 2B, C) and likely indicate sources within the marshes from contributions of fluvial-derived material, organic alkalinity, and humic acids and the breakdown of organic matter (Cai et al. 1998; Yang et al. 2015). These regions should be monitored and assessed for long-term acidification.

## Conclusions

This spatio-temporal assessment of carbonate chemistry parameters from six cruises throughout 2014 brings to light a potential hot-spot for coastal acidification within the Georgia coastal marshes. Low buffering capacity in the freshest and brackish portions of the coastal marshes is likely due to decreased pH and TA from riverine sources and increased DIC from remineralization (eDIC) of terrestrial-derived organic compounds (elevated *I*_Terr_ relative to the shelf) from the rivers and marshes. Decreased TA with elevated DIC causes the *p*CO_2_ increase in the marshes. The increased TA from warm salty offshore waters does not seem to greatly influence the state of acidification (Ω_Ar_ and Revelle factor) within the marshes during the 2014 cruises. The quantity of freshwater would be an important factor in the offshore mixing and transport of low-pH waters offshore. During 2014, with the depressed river stream flow, it is likely that the residence time of water within the marshes is greater than during higher flow years. Therefore, further study is needed to determine the impact of enhanced river stream flow in this region. This assessment is a first look at the impacts of *p*CO_2_, DIC, and TA on the state of acidification in coastal SAB marshes and suggests that these areas are potential hot-spots for acidification. Coastal acidification in SAB marshes should be further investigated over longer time scales to determine the temporal trends and short-term variability.

### Supplementary Information

Below is the link to the electronic supplementary material.

Supplementary file1 (TIF 429 KB)

## Data Availability

All data used in this research can be found in the SOCAT database and through the Georgia Coastal Ecosystems website: https://gce-lter.marsci.uga.edu/.
