# Supplementary figures and images for: Carbonate Chemistry and the Potential for Acidification in Georgia Coastal Marshes and the South Atlantic Bight, USA
Source: Estuaries Coast. 2023 Sep 11;47(1):76–90. doi: 10.1007/s12237-023-01261-3 (PMC10730646; doi:10.1007/s12237-023-01261-3)

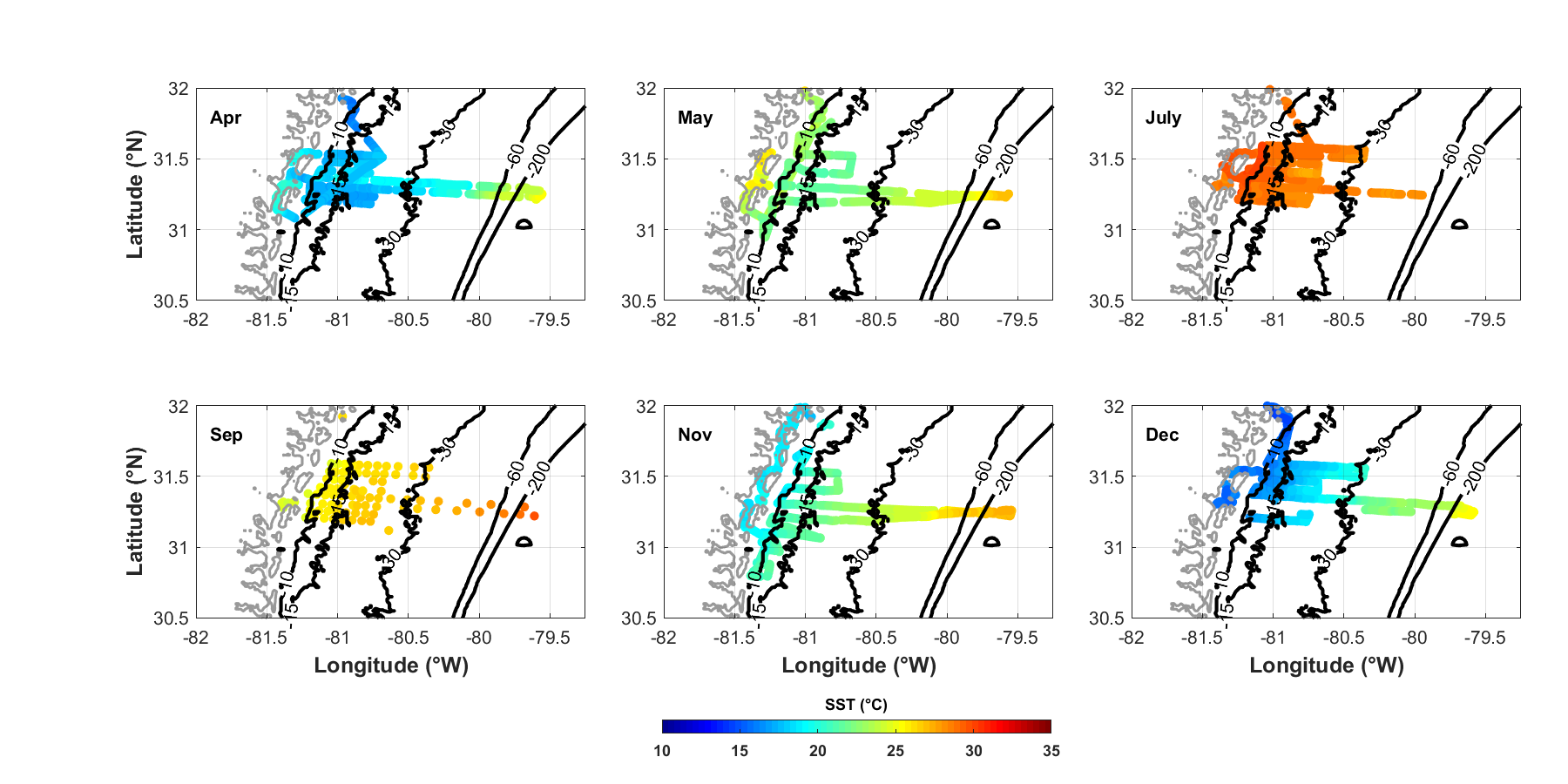

Supplement: Supplementary file 1 — Supplementary file1 (TIF 429 KB) [file 12237_2023_1261_MOESM1_ESM.tif]
